# Variation in Soil Microbial Communities Across Plantation Types in the Yellow River Floodplain of Western Shandong, China

**DOI:** 10.3390/microorganisms14061369

**Published:** 2026-06-20

**Authors:** Ke Xie, Tianxu Sun, Yongjie Miu, Ying Li, Yue Xu, Yun Cheng, Xinghui Lu

**Affiliations:** 1 College of Agriculture and Biology, Liaocheng University, Liaocheng 252000, China; 2 Shandong Territorial Spatial Planning Institute, Jinan 250014, China; 3 Key Laboratory of Forest Ecology and Environment of National Forestry and Grassland Administration, Ecology and Nature Conservation Institute, Chinese Academy of Forestry, Beijing 100091, China; 4 Liaocheng Forestry Development Center, Liaocheng 252000, China

**Keywords:** plantation, stand types, soil microbial communities, high-throughput sequencing, Yellow River floodplain

## Abstract

The Yellow River floodplain relies on plantations for ecological restoration, yet the key factors influencing soil microbial communities remain poorly elucidated. In this study, we investigated soil microbial communities under four representative stand types (*Populus tomentosa* monoculture (PP), *Salix matsudana* monoculture (PS), *Populus tomentosa*-*Robinia pseudoacacia* mixed plantation (MPR), and *Salix matsudana*-*Populus tomentosa* mixed plantation (MSP)) in this region. Using high-throughput sequencing, we compared the soil microbial community composition and diversity across stands, and combined soil physicochemical measurements to evaluate the relationships between community variation and soil factors. The results indicated that soil physicochemical properties differed significantly among stand types, except for available phosphorus. Bacterial α-diversity was highest in MPR, whereas fungal α-diversity was highest in MSP. Variation in microbial community structure (β-diversity) was primarily explained by soil organic carbon, total nitrogen, pH, water content, and electrical conductivity, as indicated by redundancy analysis and Mantel tests. The dominant bacterial phyla were Acidobacteriota, Pseudomonadota (formerly Proteobacteria), and Actinomycetota, while the dominant fungal phyla were Ascomycota, Basidiomycota, and Mortierellomycota. These findings demonstrate significant variation in soil microbial communities among plantation types and highlight the important role of soil physicochemical properties in shaping microbial community composition.

## 1. Introduction

Forests are integral to terrestrial ecosystems. They provide essential ecological services, including nutrient cycling, climate regulation, water conservation, and biodiversity maintenance [1]. However, growing human demand for ecosystem services has intensified the overexploitation and degradation of natural forests. In response, plantations have become an important supplement to global forest resources due to their key roles in ecological restoration, timber production, and carbon sequestration. Over the past decades, China has established the world’s largest area of plantation forests through a series of large-scale afforestation programs, substantially contributing to the restoration of ecologically fragile regions [2,3]. Nevertheless, compared with structurally complex natural forests, plantations often have simplified stand structures and lower ecosystem stability [4], which may compromise their long-term sustainability [5]. Located in the ecologically fragile zone of the Yellow River Basin [6], western Shandong has experienced long-term human disturbance and natural stressors, resulting in the severe depletion of natural vegetation. Consequently, plantations have become the primary tool for ecological improvement in this area [7]. The widespread dependence on plantations in this region highlights the need to ensure their long-term ecological stability. As plantations consist of structurally distinct stand types, understanding how these stand types perform under local environmental conditions is essential.

Bacteria and fungi constitute the dominant components of soil microbial communities, together accounting for more than 90% of total microbial abundance [8], yet they differ markedly in their ecological functions and responses to environmental change [9]. Fungi serve as the primary decomposers of plant residues and are particularly efficient at degrading recalcitrant compounds such as lignin and cellulose [10], whereas bacteria are generally recognized as the major drivers of soil inorganic nitrogen cycling [11]. Although these functional differences are well established, the mechanisms through which bacterial and fungal communities respond to different forest management regimes remain insufficiently understood. Previous studies have shown that stand type can influence microbial habitats through changes in litter inputs, root exudates, and soil physicochemical properties, thereby shaping microbial community composition and diversity [12,13,14,15]. For instance, mixed plantations often support greater microbial diversity and ecosystem functioning than monocultures due to enhanced nutrient cycling and resource complementarity [16].

The Yellow River floodplain of western Shandong, a representative section of the lower Yellow River, was formed through extensive alluvial deposition by the river [17]. The region is characterized by sandy loam soils with low organic matter and nutrient availability and has experienced long-term ecological degradation associated with Yellow River flooding [18]. To improve ecological conditions, large-scale plantation establishment has been implemented in western Shandong since the 1980s, leading to a plantation system dominated by *Populus tomentosa*, *Robinia pseudoacacia*, and *Salix matsudana*, which has achieved notable ecological benefits [19,20]. Despite these improvements, studies on plantation ecosystems in this region have primarily focused on soil physicochemical properties [20], carbon storage [18], and plant functional traits [21]. In contrast, soil microbial communities have received comparatively little attention. Most previous studies in this region have relied on phospholipid fatty acid (PLFA) analysis [22,23], which has limited taxonomic resolution and restricted capacity for functional inference [24]. High-throughput sequencing enables a more comprehensive characterization of bacterial and fungal community composition and provides greater taxonomic resolution for identifying microbial responses to different plantation stand types. Furthermore, systematic comparisons of microbial communities among different stand types in the floodplain are still lacking.

To elucidate how plantation structure influences belowground ecological processes, we investigated four representative stand types widely distributed in the Yellow River floodplain of western Shandong: *Populus tomentosa* monocultures, *Salix matsudana* monocultures, *Populus tomentosa*–*Robinia pseudoacacia* mixed plantations, and *Salix matsudana*–*Populus tomentosa* mixed plantations. Using high-throughput sequencing coupled with soil physicochemical analyses, we characterized variation in microbial community composition across stands and identified the soil factors underlying these patterns. Collectively, this study offers new insights into the ecological functioning of contrasting stand structures and informs species selection and management strategies for plantations in this region. Based on previous studies, we hypothesized that (1) mixed plantations would support higher microbial diversity than monoculture plantations; (2) microbial community composition would vary significantly among stand types; and (3) soil physicochemical properties would be closely associated with variation in microbial community composition.

## 2. Materials and Methods

### 2.1. Study Area

The study was conducted in the Yellow River floodplain within Liaocheng City, Shandong Province, China (35°47′–37°02′ N, 115°16′–116°32′ E). The region has a temperate monsoon climate, characterized by hot and rainy summers with concentrated precipitation, and cold, dry winters. The mean annual precipitation and temperature are 614.9 mm and 15.2 °C, respectively. The frost-free period averages 197 days per year, with a mean annual wind speed of 3.4 m s^−1^. The dominant forest types in the area are plantation forests, primarily consisting of *Populus tomentosa* Carrière, *Salix matsudana* Koidz., *Robinia pseudoacacia* L., *Fraxinus chinensis* Roxb., and *Sophora japonica* (L.) Schott.

### 2.2. Experimental Design and Soil Sample Collection

Soil sampling was conducted in November 2024 across the study area. Four stand types were selected, including two monoculture plantations (*P. tomentosa* and *S. matsudana*) and two mixed plantations (*P. tomentosa*–*R. pseudoacacia* and *S. matsudana*–*P. tomentosa*) (Table 1). For each stand type, 20 m × 20 m plots were established, with a minimum distance of 10 m between adjacent plots. In total, 54 plots were surveyed, including 17 plots in *P. tomentosa* monocultures, 13 in *S. matsudana* monocultures, 18 in *P. tomentosa*–*R. pseudoacacia* mixed plantations, and 6 in *S. matsudana*–*P. tomentosa* mixed plantations. All selected stands were mature plantations established during regional afforestation programs. The stands were of comparable age and had experienced similar site conditions, management practices, and disturbance histories.

Soil samples were collected following a standard five-point composite method (four corners and one center). Before sampling, surface litter and debris were carefully removed. Then, soil from the 0–20 cm layer was collected with sterile shovels, and the five subsamples were homogenized into a single composite sample per plot. After removing visible stones, leaves, and roots, the samples were sealed in sterile polyethylene bags, stored on ice, and transported to the laboratory [25]. In the laboratory, each sample was divided into two portions: one was placed in sterilized 5 mL polypropylene cryovials, immediately flash-frozen in liquid nitrogen, and stored at −80 °C for microbial analysis; the other was air-dried, ground, and sieved through a 2 mm mesh for the determination of soil physicochemical properties.

### 2.3. Measurements of Soil Properties

The bulk density (BD) and soil water content (SWC) were measured by the core sampling (ring knife) method and oven drying, respectively [26]. Soil electrical conductivity (EC) and pH were measured in 1:5 and 1:2.5 soil-to-water suspensions, respectively [27]. Soil organic carbon (SOC) was analyzed using the potassium dichromate oxidation titration method [27], and total nitrogen (TN) was measured using the Kjeldahl method [27]. Total phosphorus (TP), after acid digestion, and available phosphorus (AP), after NaHCO_3_ extraction, were determined using the molybdenum–antimony colorimetric method [27]. Available potassium (AK) was extracted with ammonium acetate solution and determined by flame photometry [27].

### 2.4. DNA Extraction, PCR Amplification, and Illumina Sequencing

Total genomic DNA was extracted from the soil samples using the CTAB method. DNA concentration and purity were examined on 1% agarose gels. Based on the measured concentration, DNA was diluted to 1 ng/µL with sterile water. The V4–V5 region of the bacterial 16S rRNA gene was amplified using primers 515F (5′-GTGCCAGCMGCCGCGGTAA-3′) and 907R (5′-CCGTCAATTCCTTTGAGTTT-3′) [28], while the ITS1 region of the fungal ITS rRNA was amplified using primers ITS5 (5′-GGAAGTAAAAGTCGTAACAAGG-3′) and ITS2 (5′-GCTGCGTTCTTCATCGATGC-3′) [29]. Each PCR reaction (30 µL total volume) contained 15 µL of Phusion^®^ High-Fidelity PCR Master Mix (New England Biolabs), 2 µM of each primer, and approximately 10 ng of template DNA. Thermal cycling conditions were as follows: initial denaturation at 98 °C for 1 min, followed by 30 cycles of denaturation at 98 °C for 10 s, annealing at 50 °C for 30 s, and extension at 72 °C for 30 s, with a final extension at 72 °C for 5 min. PCR products were visualized on a 2% agarose gel with 1× TAE buffer. Amplicons were mixed in equimolar concentrations and purified using the Universal DNA Purification Kit (TianGen, Beijing, China). Sequencing libraries were prepared using the NEBNext^®^ Ultra DNA Library Prep Kit for Illumina (New England Biolabs, Ipswich, MA, USA) according to the manufacturer’s protocol, and index codes were added. Library quality was assessed on an Agilent 5400 system (Agilent Technologies, Santa Clara, CA, USA), and paired-end (250 bp) sequencing was performed on an Illumina platform.

### 2.5. Bioinformatics Analysis

Raw FASTQ sequencing data were processed on the Bioincloud platform (Microeco Tech Co., Ltd., Shenzhen, China) using QIIME2 (version 2022.2). Raw reads were first imported into the QIIME2 environment and then demultiplexed, quality-filtered, trimmed, denoised, merged, and chimera-checked using the DADA2 plugin (version 1.22.0) to generate a feature table of amplicon sequence variants (ASVs) [30]. Taxonomic classification of representative ASV sequences was performed using the SILVA database (version 138.2) for bacterial 16S rRNA and the UNITE database for fungal ITS sequences, with a confidence threshold of 0.7. Alpha and beta diversity indices were calculated using the core-diversity plugin in QIIME2.

### 2.6. Statistical Analysis

Differences in soil properties and microbial (bacterial and fungal) alpha diversity among stand types were evaluated by one-way analysis of variance (ANOVA), with Duncan’s post hoc multiple comparison tests (*p* < 0.05) conducted using the “agricolae” package (version 1.3.7) [31]. Microbial beta diversity was assessed using Bray–Curtis dissimilarity-based principal coordinate analysis (PCoA) in the “vegan” package. Differences in microbial community composition among stand types were tested by permutational multivariate analysis of variance (PERMANOVA) with 999 permutations using the adonis2 function in the “vegan” package (version 2.7.1). Although plot numbers differed among stand types, PERMANOVA is considered relatively robust to moderately unbalanced sampling designs [32]. Linear discriminant analysis effect size (LEfSe; LDA > 4, *p* < 0.05) was applied to identify significantly different microbial taxa from the phylum to genus level among the stand types [33]. Prior to soil factor analyses, multicollinearity among soil variables was assessed using variance inflation factors (VIF) calculated with the “car” package (version 3.1.3). All variables had VIF values <10 and were retained for subsequent analyses (Table S1). Redundancy analysis (RDA) was performed using the “vegan” package to explore the relationships between soil properties and microbial community composition [32]. Associations between microbial community composition and soil properties were further examined using Mantel tests conducted via the “linkET” package (version 0.0.7.4). Correlations among soil properties were determined using Spearman’s rank correlation analysis. Except for LEfSe analysis, which was performed on the Wekemo Bioincloud platform (https://www.bioincloud.tech, accessed on 28 October 2025) [34] all statistical analyses and data visualizations were conducted in R version 4.4.1 [35].

## 3. Results

### 3.1. Soil Physicochemical Properties Among Stand Types

All measured soil properties varied significantly among the different stand types, except for AP (Table 2). Specifically, EC was lowest in the PP stand, while SWC and SOC were highest in the PS stand. TP was highest in the MSP stand. Among all stand types, the MPR stand had the lowest BD and the highest AK, both of which differed significantly from those of the other three stands (*p* < 0.05).

### 3.2. Soil Microbial Diversity Across Stand Types

**Figure 1:**
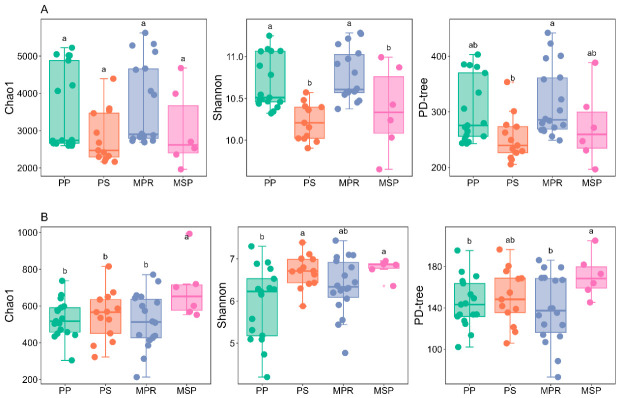
Alpha diversity indices (Chao1, Shannon, and PD-tree) of soil bacterial (**A**) and fungal (**B**) communities among different stand types. Different lowercase letters indicate significant differences. PP: *Populus tomentosa* monoculture; PS: *Salix matsudana* monoculture; MPR: *Populus tomentosa*-*Robinia pseudoacacia* mixed plantation; MSP: *Salix matsudana*-*Populus tomentosa* mixed plantation.

Bacterial alpha diversity differed among stand types for the Shannon and PD-tree indices, whereas Chao1 showed no significant difference (Figure 1A). In general, bacterial diversity tended to be higher in the MPR stand than in PS and MSP stands. In contrast, all fungal alpha-diversity indices differed significantly among stand types, with MSP consistently exhibiting the highest values (Figure 1B).

**Figure 2:**
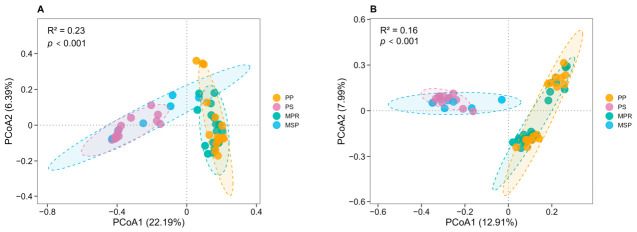
Principal coordinate analysis (PCoA) ordination based on Bray–Curtis distances for soil bacterial (**A**) and fungal (**B**) communities. Ellipses represent 95% confidence intervals. The percentages on the axes indicate the proportion of variation explained by each coordinate. PP: *Populus tomentosa* monoculture; PS: *Salix matsudana* monoculture; MPR: *Populus tomentosa*-*Robinia pseudoacacia* mixed plantation; MSP: *Salix matsudana*-*Populus tomentosa* mixed plantation.

The PCoA based on Bray–Curtis distances showed clear differences in bacterial and fungal communities among stand types (Figure 2). The first two axes explained 22.19% and 6.39% of the variation in bacterial communities (Figure 2A) and 12.91% and 7.99% of the variation in fungal communities (Figure 2B). PERMANOVA confirmed that microbial community composition differed significantly among stand types (*p* < 0.001). Pairwise comparisons showed significant differences among most stand types, whereas PS and MSP did not differ significantly (Table 3).

### 3.3. Microbial Community Composition and Differentially Abundant Taxa

**Figure 3:**
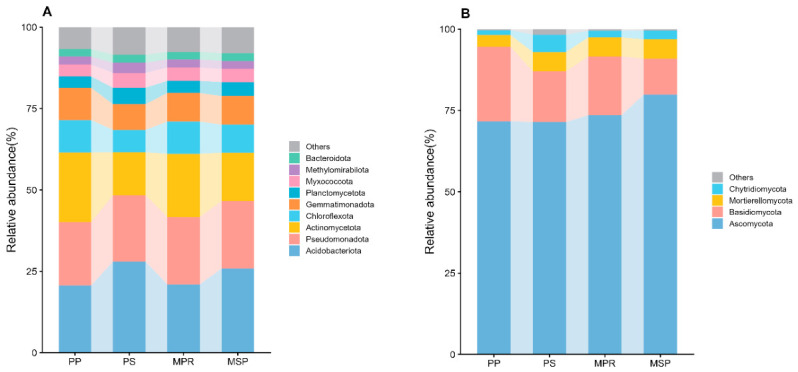
Relative abundances of dominant bacterial (**A**) and fungal (**B**) phyla among different stand types. “Others” comprises phyla with a relative abundance of less than 1%. PP: *Populus tomentosa* monoculture; PS: *Salix matsudana* monoculture; MPR: *Populus tomentosa*-*Robinia pseudoacacia* mixed plantation; MSP: *Salix matsudana*-*Populus tomentosa* mixed plantation.

A total of 49 bacterial and 13 fungal phyla were detected. Among bacteria, Acidobacteriota (21.03–28.03%), Pseudomonadota (20.35–20.78%), and Actinomycetota (13.21–21.34%) were consistently dominant across all stand types, whereas Ascomycota (71.47–79.91%) and Basidiomycota (11.02–22.87%) dominated fungal communities (Figure 3).

**Figure 4:**
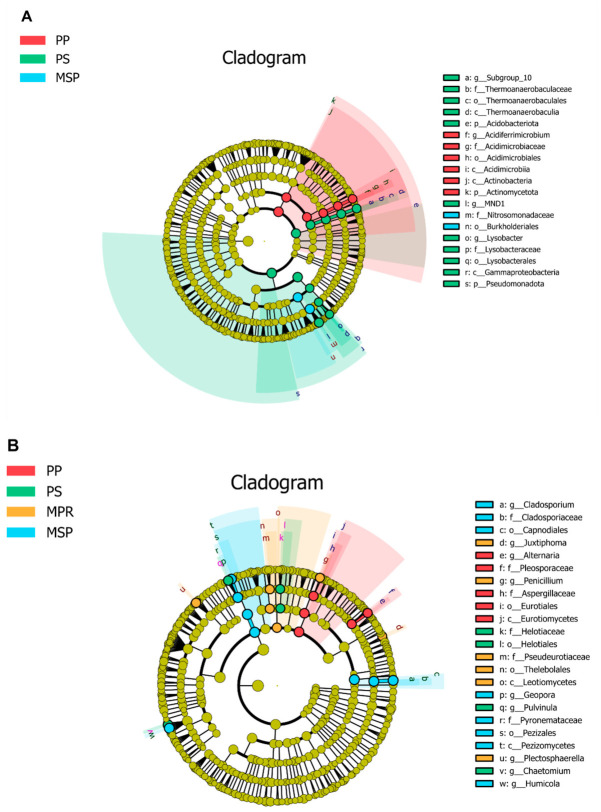
LEfSe (Linear discriminant analysis Effect Size) analysis of soil bacterial (**A**) and fungal (**B**) communities. Cladograms show the phylogenetic distribution of microbial lineages associated with different stand types. Colored nodes indicate taxa that are significantly enriched in the corresponding group (LDA score > 4), while yellow nodes indicate taxa with no significant differences. PP: *Populus tomentosa* monoculture; PS: *Salix matsudana* monoculture; MPR: *Populus tomentosa*-*Robinia pseudoacacia* mixed plantation; MSP: *Salix matsudana*-*Populus tomentosa* mixed plantation.

The LEfSe analysis identified distinct bacterial biomarkers across stand types (Figure 4A). PP was characterized by a significant enrichment of Actinomycetota-related taxa, particularly Acidimicrobiia and *Acidimicrobium*. PS showed enrichment of Pseudomonadota (Gammaproteobacteria), Acidobacteriota, and several plant-associated genera such as *Lysobacter*. MSP was mainly characterized by Burkholderiales and Nitrosomonadaceae, whereas no distinct bacterial biomarkers were detected in MPR. For fungal communities (Figure 4B), PP was enriched with Eurotiomycetes and associated taxa such as Aspergillaceae and *Alternaria*. PS was characterized by Helotiales and *Chaetomium*. MPR showed enrichment of Leotiomycetes, Pseudeurotiaceae, and *Penicillium*, while MSP was associated with Pezizomycetes and *Cladosporium*-related taxa.

### 3.4. Relationships Between Microbial Communities and Soil Properties

**Figure 5:**
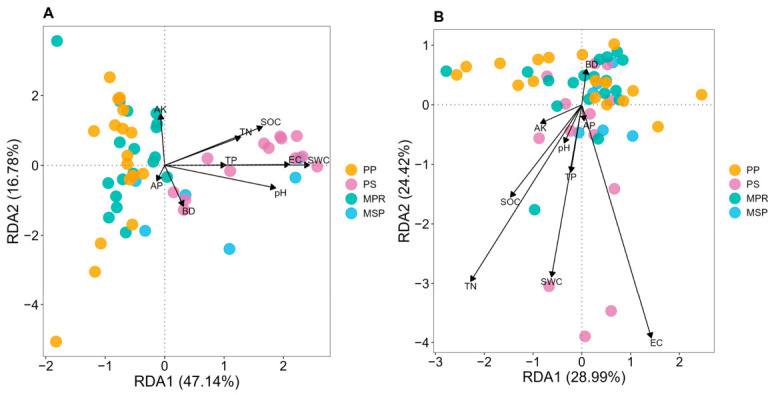
Redundancy analysis (RDA) between soil factors and soil bacterial (**A**) and fungal (**B**) community structures. BD: Bulk Density, SWC: Soil Water Content, EC: Electrical Conductivity, SOC: Soil Organic Carbon, TN: Total Nitrogen, TP: Total Phosphorus, AP: Available Phosphorus, AK: Available Potassium.

The RDA showed that soil characteristics significantly shaped the makeup of the bacterial and fungal communities, with the first two axes explaining 63.92% and 53.41% of their respective variances (Figure 5). The bacterial community was primarily affected by BD, SWC, pH, EC, SOC, TN, TP, and AK (*p* < 0.05) (Table 4), while SWC, EC, SOC, and TN (*p* < 0.05) (Table 4) exerted the greatest influence on the fungal community.

**Figure 6:**
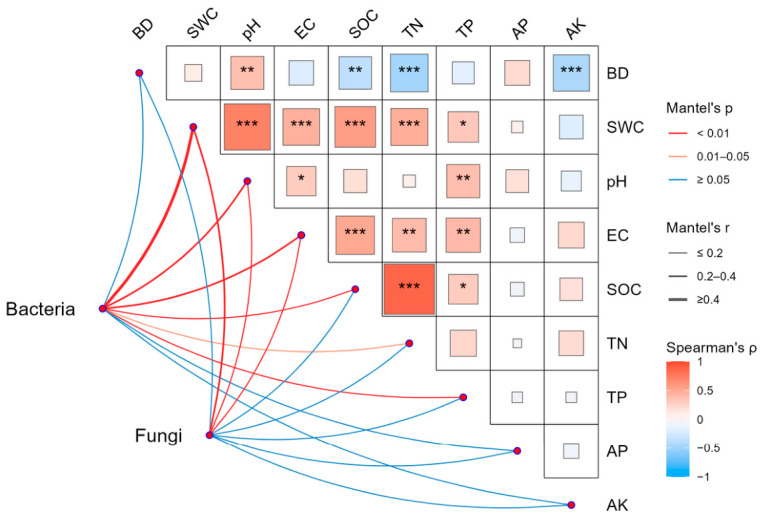
Pairwise comparisons of soil factors (heatmap) and their relationships with bacterial and fungal community composition (Mantel test). The color gradient in the heatmap indicates Spearman’s correlation coefficients between soil factors. The edge width corresponds to the Mantel’s r statistic, and the edge color denotes the statistical significance (*p* value) of the correlation. ***, **, and * denote *p* < 0.001, *p* < 0.01, and *p* < 0.05, respectively. BD: Bulk Density, SWC: Soil Water Content, EC: Electrical Conductivity, SOC: Soil Organic Carbon, TN: Total Nitrogen, TP: Total Phosphorus, AP: Available Phosphorus, AK: Available Potassium.

Mantel analysis suggested significant correlations between soil microbial communities and physicochemical properties (Figure 6). Specifically, the bacterial community was highly significantly correlated with SWC, pH, EC, SOC, TN, and TP (*p* < 0.01). In the fungal community, significant correlations were observed only with SWC, pH, and EC (*p* < 0.01), while correlations with other soil physicochemical indices were not significant.

**Figure 7:**
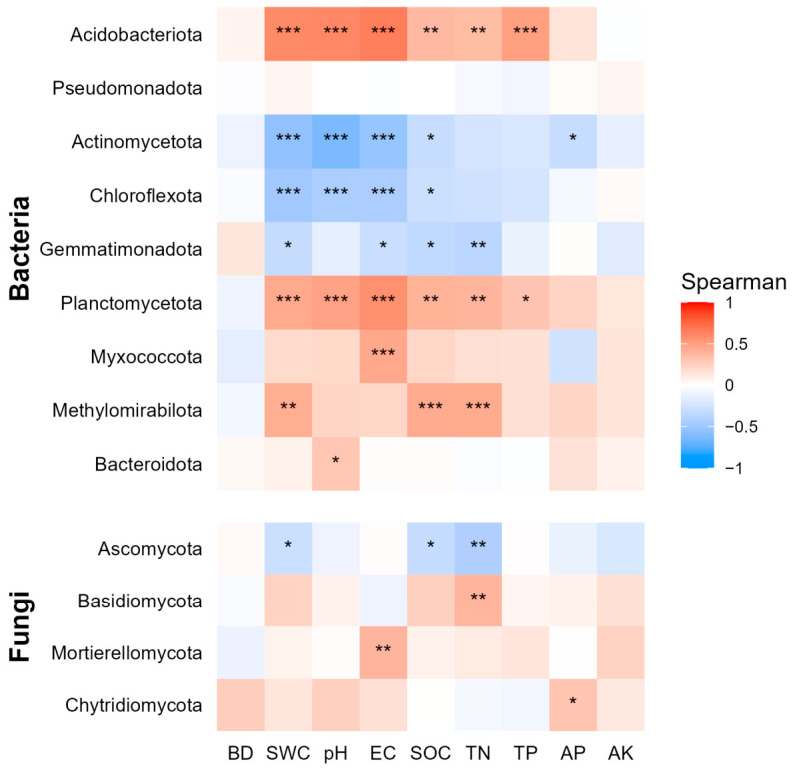
Spearman’s rank correlation heatmap between dominant microbial phyla and soil factors. The color scale indicates the correlation coefficient (r), with red representing positive correlations and blue representing negative correlations. ***, **, and * denote *p* < 0.001, *p* < 0.01, and *p* < 0.05, respectively. BD: Bulk Density, SWC: Soil Water Content, EC: Electrical Conductivity, SOC: Soil Organic Carbon, TN: Total Nitrogen, TP: Total Phosphorus, AP: Available Phosphorus, AK: Available Potassium.

Correlation analysis revealed that within the bacterial community, Acidobacteriota and Planctomycetota exhibited significant positive correlations with SWC, pH, EC, SOC, TN, and TP. Conversely, Actinomycetota and Chloroflexota showed significant negative correlations with SWC, pH, EC, and SOC (Figure 7). In the fungal community, Ascomycota was significantly negatively correlated with SWC, SOC, and TN, whereas Basidiomycota, Mortierellomycota, and Chytridiomycota displayed significant positive correlations with EC, TN, and AP, respectively (Figure 7).

## 4. Discussion

### 4.1. Variation in Soil Physicochemical Properties Among Stand Types

This study found that the MPR stand exhibited the lowest BD and the highest AK content among the four stand types, which likely reflects the enhancement of soil porosity and nutrient cycling in mixed plantations. Mixed plantations often show greater root system diversity and rhizosphere interactions, which enhance the formation of soil aggregates and increase porosity, ultimately reducing bulk density [36]. The higher AK content observed in the MPR stand may be attributed to the presence of the nitrogen-fixing species *R. pseudoacacia*. In contrast, although MSP is also a mixed plantation, it does not contain a nitrogen-fixing species and did not exhibit comparable improvements in soil properties. This suggests that the effects of mixed plantations depend not only on tree species richness per se, but also on the functional traits of the component species. The introduction of *R. pseudoacacia* may enhance nutrient inputs through biological nitrogen fixation and promote stronger belowground complementarity with *P. tomentosa* than that observed between *P. tomentosa* and *S. matsudana*. Consequently, the complementary effects on nutrient accumulation and soil structural improvement may be more pronounced in MPR than in MSP. Mixed plantations containing nitrogen-fixing species can improve soil structure and increase cation exchange capacity (CEC), thereby enhancing the soil’s ability to retain potassium ions (K^+^), which ultimately results in higher available potassium levels [37]. In addition, the PP stand exhibited the lowest EC, consistent with previous findings in poplar plantations of western Shandong [38]. As a fast-growing deep-rooted species, poplar has a high transpiration rate that substantially depletes soil moisture and may promote water percolation and salt leaching through the soil profile. This process reduces salt accumulation in the surface soil, resulting in lower EC [39]. In contrast, the PS stand had the highest SWC, likely because its litter forms a loose surface layer that does not impede rainfall infiltration [40]. Notably, although this study primarily attributes the observed differences in soil physicochemical properties to variation in stand composition, the inherent baseline characteristics of the soil itself may also contribute to these differences. Such intrinsic properties may, in turn, influence or even filter the types of tree species that can successfully establish in these stands.

### 4.2. Differences in Soil Microbial Diversity Across Stand Types

To holistically evaluate soil microbial α-diversity, we selected three widely used indices—Chao1, Shannon, and PD-tree—which respectively reflect species richness, species diversity, and phylogenetic differentiation within communities [41]. The results demonstrated that soil microbial α-diversity differed significantly among stand types (Figure 1). Specifically, bacterial diversity was significantly higher in PP and MPR stands than in PS and MSP stands, whereas fungal diversity exhibited the opposite pattern, with the highest values observed in the MSP stand. This finding indicates that bacteria and fungi respond differently to stand type-driven variations in soil environmental conditions. Previous studies have identified soil pH as a key factor influencing bacterial diversity [42], and these two factors are generally negatively correlated [43,44]. The slightly lower soil pH observed in PP and MPR stands may have contributed to their relatively higher bacterial diversity. In contrast, the highest fungal diversity observed in MSP stands may be attributed to the diverse tree species composition in mixed plantations, which provides more heterogeneous litter and plant residue inputs. These diverse organic inputs supply fungi with abundant carbon sources and complex substrates. As primary decomposers of recalcitrant organic matter, fungi can thus acquire more nutrients, thereby supporting a more diverse fungal community [45].

PCoA analysis revealed significant differences in soil microbial community composition among stand types (Figure 2), indicating that plantation composition was associated with shifts in microbial community assembly. However, the first two PCoA axes explained a relatively limited proportion of the total variation, particularly for fungal communities. This suggests that, in addition to stand type and the measured soil physicochemical properties, other factors such as litter quality, root exudates, microtopography, and stochastic ecological processes may also contribute to microbial community variation. Despite this unexplained variation, PERMANOVA results showed that no significant differences were detected in bacterial or fungal communities between the PS and MSP stands, suggesting that these stands share similar microhabitats or microbial assemblages. This similarity may be related to the presence of *S. matsudana* in both stand types together with their comparable soil physicochemical properties [46]. In contrast, although both PP and MPR stands are dominated by *P. tomentosa*, their soil microbial communities differed significantly (Table 3). This divergence may be associated with the introduction of the nitrogen-fixing species *R. pseudoacacia* in the MPR stand. Through biological nitrogen fixation, *R. pseudoacacia* can alter soil nutrient dynamics and influence microbial resource availability [47]. Moreover, its relatively high nutrient demand may accelerate phosphorus consumption and stimulate phosphatase activity under phosphorus-limited conditions [48]. Consistent with this interpretation, TP and AP contents were lower in the MPR stand than in the PP stand (Table 2). Such shifts in nitrogen and phosphorus cycling may influence microbial resource utilization and competitive interactions, thereby contributing to the observed differentiation in microbial community composition [49].

### 4.3. Ecological Implications of Community Composition and Differential Taxa

Acidobacteriota, Pseudomonadota, and Actinomycetota dominated bacterial communities across all stand types, suggesting that these taxa are well adapted to the alkaline and nutrient-limited soils characteristic of the Yellow River floodplain. Their persistence across contrasting plantation types indicates that regional edaphic conditions may exert a stronger filtering effect than stand composition. This interpretation is consistent with previous observations that these phyla possess broad ecological tolerances and can remain dominant under diverse environmental conditions [40,50,51].

The dominance of Ascomycota and Basidiomycota suggests that litter decomposition and nutrient recycling are key ecological processes in these plantation soils. Ascomycota are efficient colonizers of plant residues [51], whereas Basidiomycota specialize in the degradation of more recalcitrant compounds such as lignin [52,53]. Their coexistence therefore provides complementary functions that may contribute to ecosystem stability. The relative abundance of Ascomycota exceeding 70% may reflect the slightly alkaline soil conditions of the study area, which favor the growth of many saprotrophic fungal taxa [54].

To further identify stand-specific microbial taxa, LEfSe analysis identified several biomarkers, including Actinomycetota and Acidimicrobiia in PP stands, Pseudomonadota and Gammaproteobacteria in PS stands, Burkholderiales and Nitrosomonadaceae in MSP stands, and Leotiomycetes and Plectosphaerella in MPR stands. The differential enrichment of these taxa reflects potential functional differences in organic matter decomposition and nitrogen cycling among stand types. Specifically, Actinomycetota and Gammaproteobacteria are commonly involved in plant residue decomposition and complex organic carbon transformation [55], whereas Burkholderiales are closely associated with key nitrogen cycling processes and rhizosphere symbiosis [56]. Overall, these LEfSe-identified biomarkers clearly demonstrate the selective shaping effect of different stand types on soil microbial communities, thereby driving the enrichment of specific functional groups. This pattern is consistent with observations in other forest ecosystems [33], indicating that variations in litter quality, root exudate composition, and long-term modifications of soil physicochemical properties likely promote the formation of distinct microbial niches among stands.

### 4.4. Soil Factors Shaping Microbial Communities

Our study identifies soil physicochemical properties as the major factors associated with variation in microbial community composition across different stand types in this region. Among these, SOC, TN, pH, SWC, and EC were most strongly associated with microbial community composition. Both RDA and Mantel test results consistently showed that bacterial communities exhibited stronger correlations with these factors than fungal communities, suggesting that bacteria are generally more sensitive to changes in soil physicochemical conditions [10]. This weaker response may reflect fundamental differences in ecological strategies between bacteria and fungi. Fungi generally possess extensive hyphal networks that enable access to spatially heterogeneous resources and may therefore buffer short-term environmental fluctuations. Moreover, fungal community composition is often influenced by litter quality, plant host identity, and substrate characteristics in addition to soil chemistry, which may reduce the apparent strength of soil–fungus relationships. SOC and TN regulate substrate supply, pH influences microbial niche partitioning, SWC affects physiological activity, and EC reflects osmotic constraints [57,58,59]. Rather than acting independently, these factors likely interact to create distinct environmental filters that shape microbial community assembly across plantation types.

Many members of Acidobacteriota are generally regarded as oligotrophic bacteria characterized by relatively slow growth rates [60]. Previous studies have reported that their relative abundance is generally negatively correlated with soil pH [61,62]. However, our correlation heatmap revealed a positive relationship between Acidobacteriota and pH. Although this finding contrasts with several previous studies, it is consistent with previous findings [63]. Increasing evidence suggests that the response of Acidobacteriota to soil pH is subgroup-specific rather than uniform across the phylum [64,65]. For example, the relative abundances of Acidobacteriota subgroups 1 and 3 have been reported to be negatively correlated with soil pH, whereas subgroups 4 and 6 show positive correlations with pH [66,67]. Therefore, the overall relationship between Acidobacteriota and pH may largely depend on the composition and relative dominance of different subgroups within the community. The positive correlation observed in the present study may reflect the predominance of Acidobacteriota subgroups that are better adapted to higher pH conditions, highlighting the high ecological heterogeneity within this phylum. In addition, Chloroflexi showed negative correlations with most soil properties, which may be associated with their preference for nutrient-poor environments [68].

Our results further indicated that the relative abundance of Ascomycota was negatively correlated with soil nitrogen content, possibly due to the inhibitory effects of excessive nitrogen input on oxidative enzymes involved in lignin and aromatic compound decomposition [41]. In contrast, Basidiomycota displayed a positive correlation with total nitrogen, which may be related to their strong capacity to decompose recalcitrant organic matter and participate in nutrient cycling [69]. Thus, Basidiomycota may play a critical role in nitrogen cycling processes. However, contrasting results have been reported by [70], which may be attributed to differences in ecological context, soil organic matter composition, and nitrogen forms. Overall, distinct microbial taxa exhibited divergent responses to soil physicochemical factors, reflecting functional differentiation within microbial communities and underscoring their complex roles in ecosystem processes.

### 4.5. Limitations and Future Directions

This study provides insights into the variation of soil microbial communities across different plantation types and highlights the importance of soil physicochemical properties in shaping microbial community composition. Nonetheless, several limitations should be acknowledged. The unequal number of plots among stand types, particularly the relatively small sample size of the MSP stand, may have influenced the statistical power of some comparisons. In addition, soil samples were collected only once, and thus the observed microbial community patterns represent a single seasonal snapshot. Future studies incorporating more balanced sampling designs, repeated seasonal sampling, and additional environmental variables are needed to further validate and extend these findings.

## 5. Conclusions

This study revealed significant differences in soil physicochemical properties and microbial communities among the four plantation types in the Yellow River floodplain of western Shandong. Mixed plantations were associated with improved soil structure and nutrient availability and supported higher microbial diversity compared with monoculture plantations. Although dominant bacterial and fungal phyla were largely consistent across plantation types, microbial community composition differed significantly among stands. Soil physicochemical properties, particularly SOC, TN, pH, SWC, and EC, were strongly associated with microbial community variation, suggesting that differences in soil conditions may contribute to microbial community assembly across plantation types. These findings improve our understanding of belowground ecological processes in floodplain plantations and provide a scientific basis for plantation management and ecological restoration in the Yellow River floodplain.

## Figures and Tables

**Table 1:** Basic conditions of the four stand types.

Stand Types	Tree Species	Mean DBH (cm)	Mean Height (m)	Main Species of the Herb Layer
PP	*Populus tomentosa*	32.62 ± 10.63	26.64 ± 6.80	*Erigeron canadensis*, *Humulus scandens*, *Setaria viridis*
PS	*Salix matsudana*	21.82 ± 5.41	18.25 ± 1.65	*Artemisia argyi*, *Duchesnea indica*, *Humulus scandens*
MPR	*Populus tomentosa*	21.77 ± 12.32	17.54 ± 8.11	*Chenopodium album*, *Humulus scandens*, *Erigeron canadensis*
	*Robinia pseudoacacia*	26.61 ± 8.34	20.86 ± 6.59	
MSP	*Salix matsudana*	27.25 ± 7.22	18.36 ± 3.99	*Artemisia annua*, *Chenopodium album*, *Artemisia argyi*
	*Populus tomentosa*	25.29 ± 7.45	21.32 ± 6.36	

Note: Values are mean ± standard deviation (SD). DBH: Diameter at Breast Height, PP: *Populus tomentosa* monoculture; PS: *Salix matsudana* monoculture; MPR: *Populus tomentosa*-*Robinia pseudoacacia* mixed plantation; MSP: *Salix matsudana*-*Populus tomentosa* mixed plantation.

**Table 2:** Soil physical and chemical properties of different stand types.

Soil Properties	Stand Types			
	PP	PS	MPR	MSP
BD (g/cm^3^)	1.18 ± 0.08 a	1.18 ± 0.07 a	1.12 ± 0.05 b	1.21 ± 0.09 a
SWC (%)	11.54 ± 2.21 c	22.31 ± 3.50 a	10.98 ± 2.04 c	19.45 ± 4.43 b
pH	7.72 ± 0.13 b	7.98 ± 0.09 a	7.73 ± 0.09 b	8.02 ± 0.07 a
EC (μ/cm)	126.21 ± 15.00 b	162.38 ± 23.98 a	132.93 ± 11.27 b	150.40 ± 21.04 a
SOC (g/kg)	5.34 ± 3.38 b	7.92 ± 3.44 a	5.28 ± 1.33 b	6.19 ± 2.65 ab
TN (g/kg)	0.98 ± 0.47 b	1.43 ± 0.70 a	1.03 ± 0.35 ab	0.87 ± 0.38 b
TP (g/kg)	0.52 ± 0.30 b	0.80 ± 0.43 a	0.48 ± 0.24 b	0.95 ± 0.23 a
AP (mg/kg)	0.26 ± 0.28 a	0.22 ± 0.13 a	0.22 ± 0.08 a	0.25 ± 0.05 a
AK (mg/kg)	97.55 ± 22.51 bc	109.26 ± 18.97 b	132.38 ± 15.24 a	87.92 ± 30.81 c

Note: Values are mean ± SD. PP: *Populus tomentosa* monoculture; PS: *Salix matsudana* monoculture; MPR: *Populus tomentosa*-*Robinia pseudoacacia* mixed plantation; MSP: *Salix matsudana*-*Populus tomentosa* mixed plantation. BD: Bulk Density, SWC: Soil Water Content, EC: Electrical Conductivity, SOC: Soil Organic Carbon, TN: Total Nitrogen, TP: Total Phosphorus, AP: Available Phosphorus, AK: Available Potassium. Different lowercase letters in the same row indicate significant differences among stand types (ANOVA, *p* < 0.05, Duncan’s test).

**Table 3:** PERMANOVA test for soil microbial communities among different stand types.

Microbes	Stand Types	Pseudo-F	R^2^	*p*-Value	Adjust *p*-Value
Bacteria	PP vs. MPR	1.34	0.25	0.037	**0.044**
	PP vs. PS	9.67	0.26	0.001	**0.002**
	PP vs. MSP	3.53	0.14	0.001	**0.002**
	PS vs. MPR	9.90	0.25	0.001	**0.002**
	PS vs. MSP	1.39	0.08	0.091	0.091
	MPR vs. MSP	3.60	0.14	0.001	**0.002**
Fungi	PP vs. MPR	1.52	0.04	0.024	**0.029**
	PP vs. PS	5.37	0.16	0.001	**0.002**
	PP vs. MSP	2.76	0.12	0.001	**0.002**
	PS vs. MPR	5.07	0.15	0.001	**0.002**
	PS vs. MSP	1.01	0.06	0.440	0.440
	MPR vs. MSP	2.70	0.11	0.001	**0.002**

Note: PP: *Populus tomentosa* monoculture; PS: *Salix matsudana* monoculture; MPR: *Populus tomentosa*-*Robinia pseudoacacia* mixed plantation; MSP: *Salix matsudana*-*Populus tomentosa* mixed plantation. Values in bold indicate statistically significant differences between the two groups (*p* < 0.05).

**Table 4:** RDA results of soil microbial communities and soil properties.

Microbes	Soil Properties	RDA1	RDA2	R^2^	*p* Values
Bacteria	BD	0.2616	−0.9652	0.1192	0.0405
	SWC	0.9999	0.0051	0.6209	0.0005
	pH	0.9453	−0.3262	0.4356	0.0005
	EC	0.9999	0.0121	0.5279	0.0005
	SOC	0.8293	0.5588	0.3223	0.001
	TN	0.8427	0.5384	0.2951	0.0005
	TP	0.9999	0.0082	0.2022	0.006
	AP	−0.289	−0.9573	0.0538	0.2389
	AK	−0.048	0.9988	0.1774	0.0055
Fungi	BD	0.1557	0.9878	0.0233	0.5362
	SWC	−0.21	−0.9777	0.243	0.0015
	pH	−0.4919	−0.8707	0.034	0.4228
	EC	0.3412	−0.94	0.3559	0.0005
	SOC	−0.6864	−0.7272	0.1262	0.0315
	TN	−0.6092	−0.793	0.3311	0.0005
	TP	−0.1967	−0.9805	0.0832	0.1139
	AP	0.1723	−0.985	0.0083	0.7446
	AK	−0.9392	−0.3433	0.0511	0.2734

Note: BD: Bulk Density, SWC: Soil Water Content, EC: Electrical Conductivity, SOC: Soil Organic Carbon, TN: Total Nitrogen, TP: Total Phosphorus, AP: Available Phosphorus, AK: Available Potassium.

## Data Availability

The original contributions presented in this study are included in the article/Supplementary Materials. Further inquiries can be directed to the corresponding authors.

## Supplementary Materials

The following supporting information can be downloaded at: https://www.mdpi.com/article/10.3390/microorganisms14061369/s1, Table S1: Variance inflation factor (VIF) values of soil variables.
