# Supplementary material for: Variation in Soil Microbial Communities Across Plantation Types in the Yellow River Floodplain of Western Shandong, China
Source: Microorganisms. 2026 Jun 20;14(6):1369. doi: 10.3390/microorganisms14061369 (PMC13304021; doi:10.3390/microorganisms14061369)
Supplement: Supplementary file 1 [file microorganisms-14-01369-s001.zip › microorganisms-4338640-supplementary.pdf]

Table S1. Variance inflation factor (VIF) values of soil variables.

Variable	VIF	Variable	VIF
SWC	5.36	BD	2.30
TN	4.42	AK	1.66
SOC	3.86	TP	1.64
pH	3.38	AP	1.26
EC	2.69		

Note: BD: Bulk Density, SWC: Soil Water Content, EC: Electrical Conductivity, SOC: Soil Organic Carbon, TN: Total Nitrogen, TP: Total Phosphorus, AP: Available Phosphorus, AK: Available Potassium. All variables showed VIF values lower than 10, indicating that severe multicollinearity was not present among the soil predictors.
